# Enhancing cellulase and hemicellulase production by genetic modification of the carbon catabolite repressor gene, *creA*, in *Acremonium cellulolyticus*

**DOI:** 10.1186/2191-0855-3-73

**Published:** 2013-12-20

**Authors:** Tatsuya Fujii, Hiroyuki Inoue, Kazuhiko Ishikawa

**Affiliations:** 1 Biomass Refinery Research Center, National Institute of Advanced Industrial Science and Technology (AIST), 3-11-32 Kagamiyama, Higashi-Hiroshima, Hiroshima 739-0046, Japan

**Keywords:** Cellulase, Hemicellulase, Catabolite repression, *Acremonium cellulolyticus*

## Abstract

*Acremonium cellulolyticus* is one of several fungi that offer promise as an alternative to *Trichoderma reesei* for use in industrial cellulase production. However, the mechanism of cellulase production has not been studied at the molecular level because adequate genetic engineering tools for use in *A. cellulolyticus* are lacking. In the present study, we developed a gene disruption method for *A. cellulolyticus*, which needs a longer homologous region length. We cloned a putative *A. cellulolyticus creA* gene that is highly similar to the *creA* genes derived from other filamentous fungi, and isolated a *creA* disruptant strain by using the disruption method. Growth of the *creA* disruptant on agar plates was slower than that of the control strain. In the wild-type strain, the CreA protein was localized in the nucleus, suggesting that the cloned gene encodes the CreA transcription factor. Cellulase and xylanase production by the *creA* disruptant were higher than that of the control strain at the enzyme and transcription levels. Furthermore, the *creA* disruptant produced cellulase and xylanase in the presence of glucose. These data suggest both that the CreA protein functions as a catabolite repressor protein, and that disruption of *creA* is effective for enhancing enzyme production by *A. cellulolyticus*.

## Introduction

Lignocellulosic biomass is a promising material for use in biorefining because it contains a large amount of sugar in the form of cellulose and hemicellulose (Lynd 1996). Cellulase and hemicellulase are the two major families of enzymes that hydrolyze cellulose and hemicellulose (a lignocellulose) to monomeric sugars. Some filamentous fungi, such as *Trichoderma reesei*, secrete large amounts of cellulase and hemicellulase (Goyal et al. 1991; Krogh et al. 2004; Sehnem et al. 2006; Wen et al. 2005). The cellulases produced by fungi include three major groups of enzymes: endoglucanases, which randomly hydrolyze internal glycosidic linkages; cellobiohydrolases, which produce cellobiose from cellulose chain ends; and β-glucosidases, which convert cellobiose into glucose (Goyal et al. 1991).

The filamentous fungus, *Acremonium cellulolyticus*, which was isolated in 1982 from soil in Japan, is a cellulose-degrading organism (Yamanobe et al. 1987) and is one of several fungi that offer promise as an alternative to *T. reesei* for the industrial production of cellulase. A cellulase mixture produced by *A. cellulolyticus* is commercially sold as ‘Acremonium cellulase’ by Meiji Seika Pharma Co.. Strains TN, C-1, and CF-2612, which are cellulase hyper-producing mutants, were isolated from the wild type strain Y-94 by random mutagenesis (Fang et al. 2009; Yamanobe et al. 2003). The enzymes from *A. cellulolyticus* reportedly produce glucose more rapidly from various lignocellulosic materials than the enzymes from *T. reesei* (Fujii et al. 2009). Over 40 reports or patents related to *A. cellulolyticus* have been published, making it one of the best characterized cellulase-producing organisms. Furthermore, a genomic database (unpublished data) and transformation system for *A. cellulolyticus* (Fujii et al. 2012) have been constructed by our group. We successfully overexpressed cellulase and hemicellulase genes in this organism and constructed a starch-inducible homologous expression system (Inoue et al. 2013; Kanna et al. 2011), thus making available genetic engineering tools suitable for *A. cellulolyticus*. However, the development of these tools is not sufficient because gene targeting, such as gene disruption by homologous recombination, is difficult in *A. cellulolyticus*.

Several transcription factors have been reported as regulators of cellulase and hemicellulase gene expression in other filamentous fungi, *e.g*., XlnR/Xyr1 for genes encoding cellulase, hemicellulase, and accessory enzymes involved in xylan degradation in *Aspergillus niger* and *T. reesei* (Stricker et al. 2006; van Peij et al. 1998); Ace2 for cellulase genes in *T. reesei* (Aro et al. 2001); BglR for the β-glucosidase gene in *T. reesei* (Nitta et al. 2012); and AraR for the L-arabinose reductase gene in *A. niger* and *Aspergillus nidulans* (Battaglia et al. 2011). These transcription factors specifically regulate the expression of cellulase and hemicellulase genes. On the other hand, some transcription factors regulate a wide range of genes including cellulase and hemicellulase genes. These factors include CreA (Dowzer and Kelly 1989; Ilmen et al. 1996;: Nakari-Setälä et al. 2009; Wang et al. 2013), which is involved in catabolite repression; AreA (Lockington et al. 2002), which is involved in nitrogen source assimilation; and the Hap complex (Tsukagoshi et al. 2001), which regulates various genes. Although a number of transcription factors involved in regulating cellulase and hemicellulase gene expression in other filamentous fungi have been analyzed, the regulation of these gene expressions in *A. cellulolyticus* has not been investigated. Because *A. cellulolyticus* is an industrially important fungal species, how cellulase and hemicellulase gene expression is regulated in this organism is crucial for the development of more efficient enzyme production methods.

In the present study, we cloned a putative *creA* gene from *A. cellulolyticus* that is highly similar to the *creA* genes of other filamentous fungi and then isolated a recombinant strain in which the *creA* gene was disrupted. The length of the homologous region was important for gene disruption. Growth of the *creA* disruptant on agar plates was slower than that of the control strain, and CreA protein was found to localize in the nucleus. The production of cellulase and xylanase by the *creA* disruptant was higher than that of the control strain at both the enzyme and transcription level. Furthermore, the *creA* disruptant produced cellulase and xylanase in the presence of glucose. These data suggest that the cloned putative *creA* gene encodes a catabolite repressor protein, and that disruption of *creA* leads to enhanced enzyme productivity.

## Materials and methods

### Strains, cultures, and media

The strains used in this study are listed in Table 1. *A. cellulolyticus* YP-4 (Inoue et al. 2013), which is a uracil auxotrophic strain derived from *A. cellulolyticus* Y-94 (Yamanobe et al. 1987) (FERM BP-5826), was maintained on potato dextrose agar (PDA) (Difco, Detroit, MI) plates containing 1 g/L of uracil and 1 g/L of uridine. The transformants were maintained on MM plates (1% glucose, 10 mM NH_4_Cl, 10 mM potassium phosphate (pH 6.5), 7 mM KCl, 2 mM MgSO_4_). For measurement of enzyme activity and gene expression, the strains were cultivated in 10 mL of basic medium (24 g/L of KH_2_PO_4_, 1 g/L of Tween 80, 5 g/L of (NH_4_)_2_SO_4_, 1.2 g/L of MgSO_4_ · 7H_2_O, 0.01 g/L of ZnSO_4_ · 7H_2_O, 0.01 g/L of MnSO_4_ · 6H_2_O, 0.01 g/L of CuSO_4_ · 7H_2_O; pH 4.0) supplemented with 2 g/L of urea and 40 g/L of glycerol in 100-mL Erlenmeyer flasks at 30°C for 72 h on a rotary shaker at 230 rpm. The cells were then washed 3 times with saline, and aliquots of the washed cells were inoculated into 10 mL of basic medium supplemented with 4 g/L of urea and 10 to 50 g/L of carbon sources in 100-mL Erlenmeyer flasks which were then incubated at 30°C on a rotary shaker at 230 rpm. Cellulose (Solka Floc; Fiber Sales & Development, Urbana, OH), xylan (Birchwood xylan; SIGMA, St. Lowis, MO), glucose or glycerol were used as the carbon source.

**Table 1:** **Characteristics of the *A. cellulolyticus *** **strains and plasmids used in this study**

**Strain or Plasmid**	**Description**	**References**
Strain		
*A. cellulolyticus* Y-94	Wild type (FERM BP-5826)	Yamanobe et al. (1987)
YP-4	Uracil auxotroph mutant derived from Y-94	Inoue et al. (2013)
YDCre	YP-4 prototrophic transformant horboring pDCre2500, *creA* disruptant.	This study
YPyrF	YP-4 prototrophic transformant horboring a single copy pbs-pyrF in *pyrF* loci.	This study
YCreGFP	YP-4 prototrophic transformant harboring pCreGFP	This study
Plasmid		
pbs-pyrF	Amp^r^ PyrF^r^; pBluescript KS(+) derivative containing 2.7-kb fragment harboring *pyrF* from Y-94	Fujii et al. (2012)
pDCre2500	Amp^r^ PyrF^r^; pbs-pyrF derivative containing 2.5 kb upstream and 2.5 kb downstream regions of *creA*	This study
pDCre1000	Amp^r^ PyrF^r^; pbs-pyrF derivative containing 1.0 kb upstream and 1.0 kb downstream regions of *creA*	This study
pCreGFP	Amp^r^ PyrF^r^; pbs-pyrF derivative containing GFP and *creA* fused gene	This study

### Plasmid construction and fungal transformation

The plasmids used in this study are listed in Table 1. Plasmids used for disrupting the *creA* gene were constructed by inserting DNA fragments carrying the 5′ and 3′ regions of *creA* into the upstream and downstream regions of the *pyrF* gene in pbs-pyrF (Fujii et al. 2012). The DNA fragments carrying the 5′ regions fused with appropriate restriction sites were amplified using the primers creA 1000u-f and creA 1000u-r (for pDCre1000) or creA 2500u-f and creA 2500u-r (for pDCre2500) (Table 2), digested with *Eco*RI and *Sal*I, and ligated with pBS-pyrF which had already been spliced with the same restriction enzymes. The 3′ region of each gene was amplified using the primers creA 1000d-f and creA 1000d-r (for pDCre1000) or creA 2500d-f and creA 2500d-r (for pDCre2500) (Table 2), digested with *Xba*I and *Not*I, and inserted into the same restriction sites of the resulting plasmids to generate pDCre1000 and pDCre2500. These plasmids were digested with *Not*I before using fungal transformation. The pCreGFP plasmid used for production of the CreA-green fluorescent protein (GFP) fusion was constructed as follows. The DNA fragment encoding GFP was amplified from pGFPuv (Takara bio, Otsu, Japan) using the primers gfp-f and gfp-r (Table 2), and then digested with *Kpn*I and *Eco*RI. The *creA* DNA fragment including the promoter region was amplified using primers creAN-f and creAN-r, and then digested with *Apa*I and *Kpn*I. The resulting fragments were inserted into the *Eco*RI and *Apa*I sites of pbs-pyrF to generate pCreGFP. Fungal transformation was carried out as described previously (Fujii et al. 2012). Total fungal DNA was Southern blotted and analyzed using a DIG DNA labeling and detection kit (Roche, Basel, Switzerland) according to the manufacturer’s instructions.

**Table 2:** Nucleotide primers used in this study

**Primer**	**Nucleotide sequence**
For plasmids construction	
creA1000u-f	5′-GGGTCGACTTTACGCAGCTACGCTTAGG-3′
creA1000u-r	5′-CCGAATTCCGAACGGATATTCCTCCAAC-3′
creA1000d-f	5′-CCTCTAGAGGTGACGGGTTTAAATAAGCTG-3′
creA1000d-r	5′-CCGCGGCCGCATGCTACATGCAATCGAGTA-3′
creA2500u-f	5′-GGGTCGACTGACGGAAACGAGAATGCCG-3′
creA2500u-r	5′-GGGAATTCGCGAGGTGTAGTTGGTGTAA-3′
creA2500d-f	5′-CCTCTAGACCTTATTCACGCAACAGCGA-3′
creA2500d-r	5′-CCGCGGCCGCGTCTGGCCGAACACGTGATT-3′
gfp-f	5′-CCGTCGACATGAGTAAAGGAGAAGAACT-3′
gfp-r	5′-GGAATTCATTATTTGTAGAGCTC-3′
creAN-f	5′-CCGTCGACTAACTCCATCACGGAACCGT-3′
creAN-r	5′-CCGGTACCTAACTCCATCACGGAACCGT-3′
For quantitative PCR	
Cel5A-f	5′-CACTTGGGGTGTCGACTTCA-3′
Cel5A-r	5′-GGCAAAGGGGATACGGAAAA-3′
Cel5B-f	5′-CGACTCTGACGGGTCTGGTA-3′
Cel5B-r	5′-CTCGCTTTCCGTTGGTTTG-3′
Cel6A-f	5′-GCCGAGATCCCCTCATTTGT-3′
Cel6A-r	5′-CACGGTCAGGCAGGTCATAG-3′
Cel7A-f	5′-GGACTGCCTCCTTCAGCAAA-3′
Cel7A-r	5′-GGCGTAGTCGTCCCACAAAC-3′
Cel7B-f	5′-CCCCGGTACTTCGGTTACTT-3′
Cel7B-r	5′-CGTTGCTGATGTTGTTGTGG-3′
Xyl11B-f	5′-TGCTCTCGGTGTTGATGTTG-3′
Xyl11B-r	5′-GTGGTCTGGTAGTCGGTGGA-3′
Gapdh-f	5′-AACATCATTCCCAGCAGCAC-3′
Gapdh-r	5′-CGGCAGGTCAAGTCAACAAC-3′

### Quantitative RT-PCR

Total RNA was extracted from disrupted fungal cells. Single-stranded cDNA was synthesized and then real-time quantitative PCR proceeded as described previously (Fujii et al. 2010). Table 1 lists the gene-specific primers used. The primers were designed according to the sequence of each gene in the database: endoglucanase (*cel5A*), HV540858; endoglucanase (*cel5B*), HV540855; cellobiohydrolase II (*cel6A*), AB022429; cellobiohydrolase I (*cel7A*), E39854; endoglucanase (*cel7B*), HV540856; and xylanase (*xyl11B*), E39857. The expression of each gene was normalized against that of the glyceraldehyde dehydrogenase gene (*gpdA*). Results are shown as relative expression.

### Fluorescence microscopy

YPyrF and YCreGFP were cultured in MM medium at 30°C for 24 h, after which the cells were collected and incubated with 1 mM 4′, 6-diamidino-2-phenylindole dihydrochloride (DAPI, Lonza, Walkersville, MD) and then analyzed under a fluorescence microscope (ZEISS, Oberkochen, Germany) to determine the fluorescence excitation of GFP and DAPI.

### Other methods

Filter-paper degrading enzyme (FPase) and xylanase activities were measured as previously described (Fujii et al. 2009). The concentration of soluble protein was determined using the method of Lowry et al. (Lowry et al. 1951). The glucose concentration was determined using an HPLC system equipped with an RI-2031 Plus refractive index detector (Jasco, Tokyo, Japan) and an Aminex HPX-87P column (Bio-Rad, Hercules, CA) fitted with a Carbo-P micro-guard cartridge (Bio-Rad). The mobile phase was double-deionized water, the flow rate was 1.0 mL/min, and the column temperature was 80°C.

### Accession numbers

The nucleotide and amino acid sequences of *creA* and *gpdA* from Y-94 are to appear in the GenBank/EMBL/DDBJ nucleotide database under accession nos. AB847424 and AB847425, respectively.

## Results

### Characterization of the putative *creA* gene

**Figure 1:**
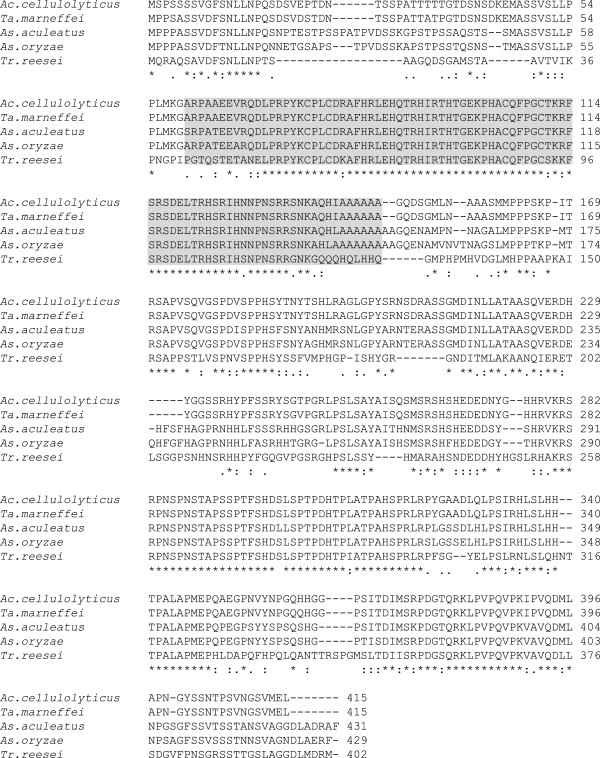
**A comparison of the deduced amino acid sequences for CreA protein from *A. cellulolyticus *Y-94, *Talaromyces marneffei, Aspergillus aculeatus, Aspergillus oryzae, *and *Trichoderma reesei.*** The alignment was created using ClustalW2 (http://www.ebi.ac.uk/Tools/msa/clustalw2/). The zinc-finger domain is indicated by gray shadowing.

First, we searched the *A. cellulolyticus* Y-94 genome database for a putative *creA* gene (unpublished data). A 1248 bp nucleotide sequence was found that encodes a 415 amino acid protein. A database search revealed that the amino acid sequence of this protein was similar to that of the CreA protein of *Talaromyces marneffei* (XP_002152134), *Aspergillus acleatus* (O94166), *Aspergillus oryzae* (AAK11189), and *T. reesei* (BAA09784), at 98%, 76%, 75%, and 53% identity, respectively (Figure 1). The predicted protein contains a zinc-finger domain that is conserved among the *creA* proteins (Figure 1). These data suggest that the nucleotide sequence we identified encodes an *A. cellulolyticus* Y-94 ortholog of the *creA* gene.

### Isolation of a *creA* disruptant strain

**Figure 2:**
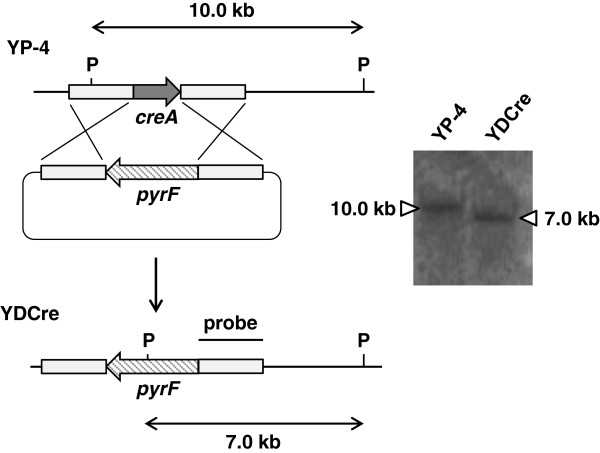
**Disruption of the *creA *gene in *A. cellulolyticus.*** The strategy for homologous recombination into the *creA* locus to construct *creA* gene disruptants is shown. Total DNA was isolated and digested with *Pst*I before Southern blotting.

Isolation of a strain with a disrupted *creA* gene is essential for analysis of the gene’s function. Gene disruption was carried out by homologous recombination, and we confirmed that *creA* was replaced with the *pyrF* marker, as shown in Figure 2. When pDCre1000 (with the homologous region length set to 1,000 bp) was introduced into YP-4, no gene-disrupted strains were isolated from the 300 transformants showing restored uracil auxotrophy (Table 3). This result was consistent with our previous finding that exogenous DNA is commonly integrated into the *A. cellulolyticus* genome with nonhomologous recombination (Fujii et al. 2012). On the other hand, when pDCre2500 (with the homologous region length set to 2,500 bp) was introduced, 19 gene-disrupted strains were obtained from only 71 transformants (Table 3). Southern blotting of total DNA from the YP-4 and YDCre (*creA* disruptant) strains revealed a 10 kb *Pst*I DNA fragment specific for YPyrF *creA* but not for YDCre *creA*. YDCre generated a 7 kb band, indicating a deletion of the *creA* gene (Figure 2). These data indicate that the length of the homologous regions is important for gene disruption in *A. cellulolyticus*. To our knowledge, YDCre is the first *A. cellulolyticus* gene-disrupted strain produced by homologous recombination. We also constructed YPyrF as a control strain, which harbors a single copy of pbs-pyrF in the *pyrF* locus of the YP-4 genome (data not shown).

**Table 3:** **Efficiency of *A. cellulolyticus creA *** **gene disruption**

**Introducing plasmids**	**Number of transformants**	**Number of *creA *** **disruptants**	**Gene disruption efficiency (%)**
pDCre1000	300	0	0
pDCre2500	71	19	27

**Figure 3:**
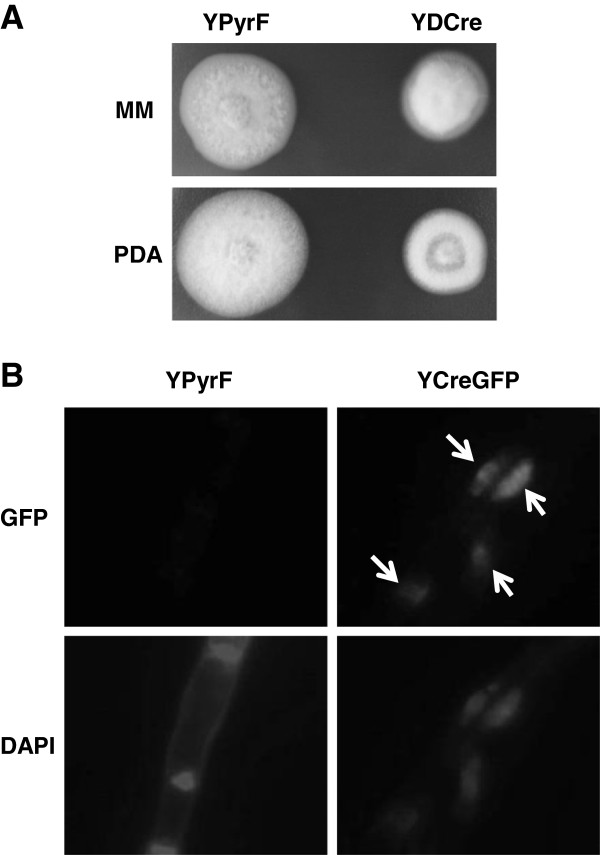
**Characterization of the *creA *disrupted strain and the *creA***-**GFP fused gene strain. A**, Photographs showing YPyrF and YDCre cells cultured on MM and PDA media. **B**, YPyrF and YCreGFP were cultured in MM medium for 1 day, and then observed under a fluorescence microscope. Fluorescence emitted by GFP is indicated by an arrowhead.

Growth of YDCre on MM and PDA medium was slower than that of YPyrF (Figure 3A), which is consistent with the growth rate of *creA* disruptants of other filamentous fungi. Cellular localization of CreA protein was investigated by observation of CreA-GFP fusion protein fluorescence. Figure 3B shows that fluorescence was emitted by GFP in DAPI-stained nuclei, indicating that CreA protein was localized in the nucleus. These data confirm that the cloned gene encodes CreA protein.

### The role of *creA* in cellulase and hemicellulase production

**Figure 4:**
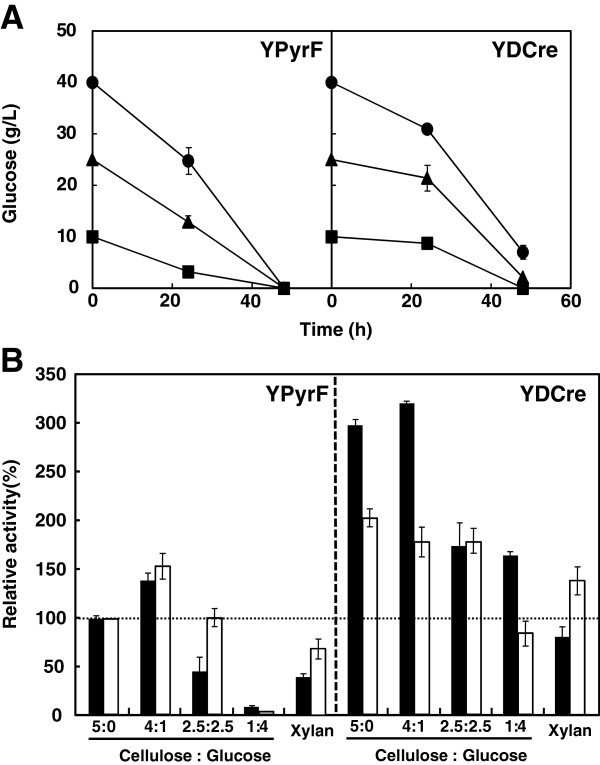
**Cellulase and xylanase activities of the strains under various culture conditions. A**, Glucose consumption. The strains were cultured in cellulose- and glucose-containing medium. Initial cellulose and glucose concentrations were 10 and 40 g/L (circles), 25 and 25 g/L (triangles), and 40 and 10 g/L (squares), respectively. **B**, FPase and xylanase activities of the strains under various culture conditions. Initial cellulose and glucose concentrations were 50 and 0 g/L (5:0), 40 and 10 g/L (4:1), 25 and 25 g/L (2.5:2.5), and 10 and 40 g/L (1:4), respectively. Initial xylan concentration was 50 g/L. Black bars, FPase; white bars, xylanase. Data are presented as the mean of three experiments.

YPyrF and YDCre were cultured in medium containing cellulose and glucose, and the glucose concentration in the culture supernatant was then measured. Although YPyrF consumed all of the glucose within 48 h, irrespective of the starting concentration, some glucose remained in the YDCre supernatants after 48 h of cultivation (Figure 4A).

Next, we measured the activity of the enzymes FPase and xylanase in the culture supernatant after 48 h of cultivation under various conditions. The activity in the YPyrF supernatant when the cells were grown only on cellulose (5:0) was taken as 100% (Figure 4B). The relative FPase activity in YpyrF supernatants was 142% (cellulose:glucose = 4:1), 45% (2.5:2.5), and 5% (1:4), whereas the relative xylanase activity was 155% (4:1), 101% (2.5:2.5), and 3% (1:4) (Figure 4B). When the glucose concentration was high (1:4), the FPase and xylanase activities of YPyrF were very low, indicating that cellulase and xylanase production were repressed by glucose. The relative FPase activity in YDCre supernatants was 301% (5:0), 324% (4:1), 173% (2.5:2.5), and 168% (1:4), whereas the relative xylanase activity was 201% (5:0), 176% (4:1), 175% (2.5:2.5), and 85% (1:4) (Figure 4B). The activity of both enzymes was higher than that of the YPyrF enzymes under all conditions tested. Furthermore, although the activity of both enzymes in the YPyrF culture was very low at the highest glucose:cellulose ratio examined, their activity was much higher in the YDCre culture grown under the same conditions. When xylan was used as the sole carbon source, YDCre produced higher FPase and xylanase activities than YPyrF (Figure 4B). These data suggest that *creA* is involved in repression of cellulase and xylanase production.

**Figure 5:**
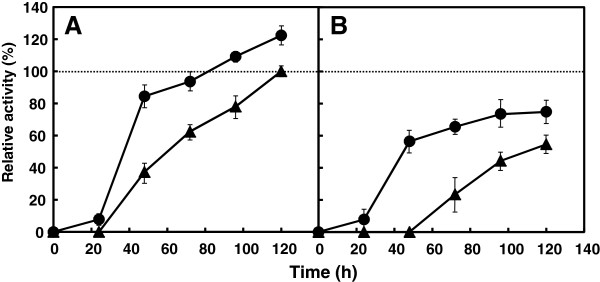
**Time**-**dependent cellulase activity of the strains.** Strains were cultured in medium containing 50 g/L of cellulose **(A)**, or 10 g/L of cellulose and 40 g/L of glucose **(B)**. Circles, YDCre; triangles, YPyrF. Data are presented as the mean of three experiments.

We then examined FPase production by YDCre and YPyrF in time-course experiments (Figure 5). When cellulose was used as the sole carbon source, YDCre showed 1.25-fold higher FPase activity than YPyrF after 120 h of cultivation. When the strains were cultured under cellulose and glucose conditions (cellulose:glucose = 1:4), YDCre exhibited 1.3-fold higher FPase activity than YPyrF after 120 h of cultivation. Furthermore, YDCre exhibited significantly higher FPase activity than did YPyrF in the early stages of culture (after 48 h of cultivation) under both conditions, indicating that cellulase production is induced more rapidly in YDCre than in YPyrF. These data suggest that disruption of *creA* in *A. cellulolyticus* results in effective cellulase production.

### The role of creA in cellulase and hemicellulase gene expression

**Figure 6:**
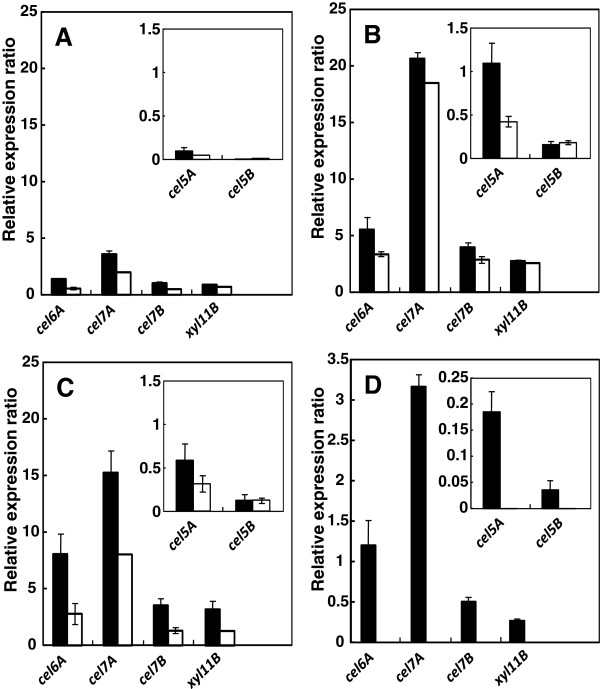
**Expression of cellulase and xylanase genes**. Strains were cultured in medium containing 50 g/L of cellulose for 24 h **(A)**, 72 h **(B)**, 120 h **(C)**, or in 10 g/L of glucose and 40 g/L of cellulose for 48 h **(D)**. Expression levels are shown relative to that of *gpdA* as an internal control. Black bars, YDCre; white bars, YPyrF. Data are presented as the mean of three experiments.

The levels of cellulase and xylanase gene expression in YDCre and YPyrF cultured for 24, 72, and 120 h were measured by quantitative RT-PCR (Figure 6A-C). Expression of cellulase and xylanase genes was observed after 24 h (*e.g*., relative expression ratio of *cel7A*, encoding cellubiohydrolase I, of YpyrF; 2.0), reached their maximum levels by 72 h (18.5), and had decreased by 120 h (8.1) in both strains (Figure 6A-C). The gene *cel7A* exhibited the highest expression level among the analyzed genes, followed by *cel6A* (cellobiohydrolase II) and *cel7B* (endoglucanase). Gene expression was much higher in YDCre than in YPyrF (with the exception of *cel5B*, endoglucanase, at 120 h), which is consistent with the result showing that cellulase and xylanase enzymatic activity are higher in YDCre than in YPyrF. These data indicate that cellulase and xylanase production in YDCre is regulated at the transcriptional level. When the strains were cultured in medium containing cellulose and glucose (culturing for 48 h; some glucose remained in the YDCre supernatants, Figure 4A), no expression was detected in YPyrF for any of the genes analyzed. In contrast, expression of all the genes was detected in YDCre (Figure 6D). These data indicate that expression of the genes analyzed in this study is repressed by glucose addition and that this repression is abolished by disruption of *creA*.

## Discussion

In this study, we characterized the function of the *A. cellulolyticus* gene, *creA*. The deduced amino acid sequence of CreA showed that it is highly similar to CreAs produced by other filamentous fungi. In addition, CreA was found to localize in the nucleus of *A. cellulolyticus*. Strain YDCre showed higher cellulase and xylanase activity than strain YPyrF, and repression of enzyme activity by glucose was abolished in YDCre. These activities were regulated at the transcription level; taken together, these data suggest that CreA is a transcription factor involved in carbon catabolite repression. The present study is the first report demonstrating improved enzyme production following modification of transcriptional regulation in *A. cellulolyticus*.

In our previous study, we noticed that exogenous DNA can be commonly integrated into the *A. cellulolyticus* genome through non-homologous recombination (Fujii et al. 2012). In fact, introduction of pDCre1000 into YP-4 precluded the generation of a *creA* disruptant, indicating that pDCre1000 was integrated through non-homologous recombination. However, introduction of pDCre2500 into YP-4 resulted in the isolation of *creA* disruptants with satisfactory frequency (27%). Furthermore, we obtained a transformant that carried pbs-pyrF in the *pyrF* locus by introducing digested pbs-pyrF (*pyrF* internal digestion) (Fujii et al. 2012). These data suggest that rigorous conditions for introducing DNA are necessary for gene targeting in *A. cellulolyticus*; however, a future study will make rapid progress by using a the gene disruption strategy based on the present study.

The cellulase and xylanase activities (Figures 4 and 5) and the levels of the corresponding mRNAs produced (Figure 6) were higher in YDCre than in YPyrF. Enhanced enzyme activity and gene expression were observed in both glucose-containing medium and medium containing only cellulose. Furthermore, the higher cellulase and xylanase activities of YDCre were observed when the strains were cultured with xylan. These data suggest that the repression involving *creA* is responsive to various carbon sources. Enzyme production in a *T. reesei*, *creA*-knockout strain was shown to be higher than in the parental strain under certain conditions (Nakari-Setälä et al. 2009), which is consistent with our results described above.

In other filamentous fungi, CreA protein regulates gene expression by binding to the promoter region (Ilmen et al. 1996). Three binding sequences of CreA protein (5′-SYGGRG-3′) in other fungi were identified in a 1300 bp upstream region of *cel7A* of *A. cellulolyticus* (data not shown). Furthermore, the binding sequences were found in the upstream region of other genes analyzed in Figure 6 (data not shown). These data imply that the CreA protein of *A. cellulolyticus* represses gene expression by binding to the promoter regions. In *T. reesei*, transcription of Xyr1, which is currently considered a main inducer of cellulase and xylanase production, and of Ace1, which is a specific repressor for cellulase and hemicellulase production, were repressed by carbon catabolite repression involving CreA protein (Mach-Aigner et al. 2008; Portnoy et al. 2011). These data suggest that CreA protein of *T. reesei* regulates not only cellulase and hemicellulase genes but also their transcription factors. CreA protein of *A. cellulolyticus* may regulate other transcription factors involving cellulase and hemicellulase production as *T. reesei*; however, no transcription factors other than CreA of *A. cellulolyticus* have been investigated. Hence, further experiments, such as the identification of other transcription factors, are needed to address the mechanism of regulation of cellulase and hemicellulase production by *A. cellulolyticus*.

The results obtained in this study strongly indicate that disruption of *creA* leads to elevated cellulase and hemicellulase production in *A. cellulolyticus*. We are currently analyzing other transcription factors that are expected to regulate the production of these enzymes, and intend to further improve cellulase and hemicellulase production by *A. cellulolyticus* by modifying these factors.

## Competing interests

The authors declare that they have no competing interests.

## References

[B1] Aro N Saloheimo A Ilmen M Penttilä M AceII, a novel transcription activator involved in regulation of cellulase and xylanase genes of *Trichoderma reesei* J Biol Chem 2001 3 24309 24314 10.1074/jbc.M003624200 11304525

[B2] Battaglia E Hansen S Leendertse A Madrid S Mulder H Nikolaev I de Vries R Regulation of pentose utilisation by AraR, but not XlnR, differs in *Aspergillus nidulans* and *Aspergillus niger* Appl Microbiol Biotechnol 2011 3 387 397 10.1007/s00253-011-3242-2 21484208 PMC3125510

[B3] Dowzer C Kelly J Cloning of the *creA* gene from *Aspergillus nidulans*: a gene involved in carbon catabolite repression Curr Genet 1989 3 457 459 10.1007/BF00376804 2673558

[B4] Fang X Yano S Inoue H Sawayama S Strain improvement of *Acremonium cellulolyticus* for cellulase production by mutation J Biosci Bioeng 2009 3 256 261 10.1016/j.jbiosc.2008.11.022 19269588

[B5] Fujii T Fang X Inoue H Murakami K Sawayama S Enzymatic hydrolyzing performance of *Acremonium cellulolyticus* and *Trichoderma reesei* against three lignocellulosic materials Biotechnol Biofuels 2009 3 24 10.1186/1754-6834-2-24 19796378 PMC2761304

[B6] Fujii T Iwata K Murakami K Yano S Sawayama S Isolation of uracil auxotrophs of the fungus *Acremonium cellulolyticus* and the development of a transformation system with the *pyrF* gene Biosci Biotechnol Biochem 2012 3 245 249 10.1271/bbb.110498 22313749

[B7] Fujii T Murakami K Sawayama S Cellulase hyperproducing mutants derived from the fungus *Trichoderma reesei* QM9414 produced large amounts of cellulase at the enzymatic and transcriptional levels Biosci Biotechnol Biochem 2010 3 419 422 10.1271/bbb.90655 20139594

[B8] Goyal A Ghosh B Eveleigh D Characteristics of fungal cellulases Bioresour Technol 1991 3 37 50 10.1016/0960-8524(91)90098-5

[B9] Inoue H Fujii T Yoshimi M Taylor LE 2nd Decker SR Kishishita S Nakabayashi M Ishikawa K Construction of a starch-inducible homologous expression system to produce cellulolytic enzymes from *Acremonium cellulolyticus* J Ind Microbiol Biotechnol 2013 3 823 830 10.1007/s10295-013-1286-2 23700177

[B10] Ilmen M Onnela ML Klemsdal S Keranen S Penttilä M Functional analysis of the cellobiohydrolase I promoter of the filamentous fungus *Trichoderma reesei* Mol Gen Genet 1996 3 303 314 9003317 10.1007/pl00008597

[B11] Kanna M Yano S Inoue H Fujii T Sawayama S Enhancement of β-xylosidase productivity in cellulase producing fungus *Acremonium cellulolyticus* AMB Express 2011 3 15 10.1186/2191-0855-1-15 21906369 PMC3222308

[B12] Krogh KB Mørkeberg A Jørgensen H Frisvad JC Olsson L Screening genus *Penicillium* for producers of cellulolytic and xylanolytic enzymes Appl Biochem Biotechnol 2004 3 389 401 10.1385/ABAB:114:1-3:389 15054266

[B13] Lockington RA Rodbourn L Barnett S Carter CJ Kelly JM Regulation by carbon and nitrogen sources of a family of cellulases in *Aspergillus nidulans* Fungal Genet Biol 2002 3 190 196 10.1016/S1087-1845(02)00504-2 12409103

[B14] Lowry OH Rosebrough NJ Farr AL Randall RJ Protein measurement with the folin-phenol reagent J Biol Chem 1951 3 265 275 14907713

[B15] Lynd LR Overview and evaluation of fuel ethanol from cellulosic biomass: technology, economics, the environment, and policy Annu Rev Energy Environ 1996 3 403 465 10.1146/annurev.energy.21.1.403

[B16] Mach-Aigner AR Pucher ME Steiger MG Bauer GE Preis SJ Mach RL Transcriptional regulation of *xyr1*, encoding the main regulator of the xylanolytic and cellulolytic enzyme system in *Hypocrea jecorina* Appl Environ Microbiol 2008 3 6554 6562 10.1128/AEM.01143-08 18791032 PMC2576687

[B17] Nakari-Setälä T Paloheimo M Kallio J Vehmaanperä J Penttilä M Saloheimo M Genetic modification of carbon catabolite repression in *Trichoderma reesei* for improved protein production Appl Environ Microbiol 2009 3 4853 4860 10.1128/AEM.00282-09 19447952 PMC2708423

[B18] Nitta M Furukawa T Shida Y Mori K Kuhara S Morikawa Y Ogasawara W A new Zn(II)2Cys6-type transcription factor BglR regulates β-glucosidase expression in *Trichoderma reesei* Fungal Genet Biol 2012 3 388 397 10.1016/j.fgb.2012.02.009 22425594

[B19] Portnoy T Margeot A Seidl-Seiboth V Le Crom S Ben Chaabane F Linke R Seiboth B Kubicek CP Differential regulation of the cellulase transcription factors XYR1, ACE2, and ACE1 in *Trichoderma reesei* strains producing high and low levels of cellulase Eukaryot Cell 2011 3 262 271 10.1128/EC.00208-10 21169417 PMC3067402

[B20] Sehnem NT Bittencourt LR Camassola M Dillon AJP Cellulase production by *Penicillium echinulatum* on lactose Appl Microbiol Biotechnol 2006 3 163 167 10.1007/s00253-005-0251-z 16408174

[B21] Stricker A Grosstessner-Hain K Würleitner E Mach R Xyr1 (xylanase regulator 1) regulates both the hydrolytic enzyme system and D-xylose metabolism in *Hypocrea jecorina* Eukaryot Cell 2006 3 2128 2137 10.1128/EC.00211-06 17056741 PMC1694815

[B22] Tsukagoshi N Kobayashi T Kato M Regulation of the amylolytic and (hemi-)cellulolytic genes in aspergilli J Gen Appl Microbiol 2001 3 1 19 10.2323/jgam.47.1 12483563

[B23] van Peij N Gielkens M de Vries R Visser J de Graaff L The transcriptional activator XlnR regulates both xylanolytic and endoglucanase gene expression in *Aspergillus niger* Appl Environ Microbiol 1998 3 3615 3619 9758775 10.1128/aem.64.10.3615-3619.1998 PMC106473

[B24] Wang S Liu G Yu J Tian S Huang B Xing M RNA interference with carbon catabolite repression in *Trichoderma koningii* for enhancing cellulase production Enzyme Microb Technol 2013 3 104 109 10.1016/j.enzmictec.2013.04.007 23769310

[B25] Wen Z Liao W Chen S Production of cellulase/β-glucosidase by the mixed fungi culture *Trichoderma reesei* and *Aspergillus phoenicis* on dairy manure Process Biochem 2005 3 3087 3094 10.1016/j.procbio.2005.03.044 15917591

[B26] Yamanobe T Mitsuishi Y Takasaki Y Isolation of a cellulolytic enzyme producing microorganism, culture conditions and some properties of the enzymes Agric Biol Chem 1987 3 65 74 10.1271/bbb1961.51.65

[B27] Yamanobe T Okuda N Oouchi K Suzuki K This patent includes isolation of A. cellulolyticus strain C-1, which is cellulase hyper-producing mutant strain derived from strain Y-94 2003 Japanese patent: Japanese patent 2003–135052 13 May 2003

